# Role of K63-linked ubiquitination in cancer

**DOI:** 10.1038/s41420-022-01204-0

**Published:** 2022-10-06

**Authors:** Liangzi Cao, Xiaofang Liu, Bowen Zheng, Chengzhong Xing, Jingwei Liu

**Affiliations:** Department of Anus and Intestine Surgery, First Affiliated Hospital of China Medical University, Shenyang, Liaoning Province China

**Keywords:** Ubiquitylation, Ubiquitylated proteins

## Abstract

Ubiquitination is a critical type of post-translational modifications, of which K63-linked ubiquitination regulates interaction, translocation, and activation of proteins. In recent years, emerging evidence suggest involvement of K63-linked ubiquitination in multiple signaling pathways and various human diseases including cancer. Increasing number of studies indicated that K63-linked ubiquitination controls initiation, development, invasion, metastasis, and therapy of diverse cancers. Here, we summarized molecular mechanisms of K63-linked ubiquitination dictating different biological activities of tumor and highlighted novel opportunities for future therapy targeting certain regulation of K63-linked ubiquitination in tumor.

## Facts

- Ubiquitination is a critical type of post-translational modifications.
- K63-linked ubiquitination regulates interaction, translocation, and activation of proteins.
- K63-linked ubiquitination controls initiation, development, invasion, metastasis, and therapy of diverse cancers.
- Increasing technologies and comprehensive understanding of ubiquitination signal suggest promising therapeutic strategies to improve tumor therapy.

## Open questions

- The role of K63-linked ubiquitination in the initiation, development, and metastasis of cancer was not clearly clarified.
- What are the molecular mechanisms of K63-linked ubiquitination dictating different biological activities of tumor?
- Is it a promising strategy in clinical cancer treatment targeting ubiquitinated proteins and sites?

## Introduction

As one of the most essential regulation of proteins, post-translational modifications (PTMs) represent the residue modifications of certain amino acid, which determine functions of proteins in various aspects [1]. PTMs mainly involve ubiquitination, phosphorylation, acetylation, methylation, glycosylation, and SUMOylation, which regulate multiple biological progresses including proliferation, death, differentiation, and cell cycle [2]. Ubiquitination is the connection of ubiquitin, a molecule consisted with 76-amino acid, with the substrate protein or itself in covalent form via its C-terminus [3]. According to the number of connected ubiquitin molecules, ubiquitination can be classified into mono-ubiquitination (modified by a single ubiquitin molecule) and poly-ubiquitination (modified by a ubiquitin chain) [4]. From a functional perspective, mono-ubiquitination often alters the interaction, localization, and transport of protein substrates, while poly-ubiquitination is mainly related with proteasome-dependent degradation, activity and translocation of the substrates [5]. As for poly-ubiquitination, ubiquitin molecule can be linked each other through seven different receptor sites, since ubiquitin molecules contain seven sites of lysine (K6, K11, K27, K29, K33, K48, and K63) [6]. K48-linked polyubiquitin chains are usually related with the proteolysis of certain substrates through the ubiquitin-proteasome system [7]. K63-linked polyubiquitin chains, however, modulate the activity, interaction or intracellular trafficking of tagged proteins, participating in diverse biological procedures [3].

A number of properties of protein could be affected by K63-linked ubiquitination such as protein-protein interaction, translocation, and activation. For example, the K63-linked ubiquitination of β-catenin is regulated by RNF8, which promotes its nuclear translocation and further oncogenic activity [8]. In addition, K63-linked polyubiquitination of ERK controlled by TRIM15 and CYLD determines its interaction with and activation by MEK [9]. Various physiological and pathological processes could be influenced by K63-linked ubiquitination. For instance, K63-linked ubiquitination of TBK1, regulated by E3 ubiquitin ligase RNF128, facilitates innate immunity [10]. K63-linked ubiquitination of YAP induced by IL-1b-TRAF6 signaling in macrophages leads to increased YAP stability and nuclear entry, resulting in pro-inflammatory gene expression and subsequently atherosclerosis [11]. Integrin α3β1 controls Akt K63-linked polyubiquitination in a TRAF6-dependent manner, thus modulating the development of kidney collecting duct [12]. Importantly, K63-linked ubiquitination influence different processes of cancer, including tumorigenesis, development, metastasis and therapy. In this review, we summarized different aspects of tumor biological activities in relation to K63-linked ubiquitination as well as their molecular mechanisms and potential future applications.

## K63-linked ubiquitination and tumorigenesis

Tumorigenesis is a gradual process by which normal cells develop into tumors, which involves multistage reactions and the accumulation of key mutations. A number of signaling pathways are often altered including PI3K/AKT signaling, Wnt/β-Catenin signaling, SAPK/JNK signaling, Hippo Signaling in the tumor initiation [13].

### PI3K/Akt signaling

PI3K/AKT signaling pathway participates in the regulation of multiple biological processes of proliferation, metabolism, growth, transcription, and protein synthesis [14]. Akt regulates proliferation of cells by mTOR signaling and phosphorylating CDK inhibitors p21 and p27. As a serine/threonine kinase, AKT is activated by PI3K or phosphoinositide-dependent kinases (PDK), which is frequently dysregulated in multiple diseases of diabetes, cardiovascular diseases, and cancer [15].

The K63-linked polyubiquitination of Akt was found to regulate tumorigenesis. The K63-linked ubiquitination Akt and its activation is affected by E3 ligase activity of Skp2 SCF complex, which is responsible for subsequent oncogenesis [16]. Furthermore, SETDB1 methylates Akt and facilitates the K63-linked ubiquitination as well as activation of Akt, resulting in tumor initiation [17]. In addition, E3 ubiquitin ligase TRAF2 and deubiquitinating enzyme OTUD7B control a K63-linked polyubiquitination switch of GβL that modulates homeostasis and activation of mTORC2/AKT signaling [18].

### Wnt/β-catenin

The Wnt/β-catenin pathway modulates the pluripotency of stem cell and affects how cells differentiate during development [19]. The on or off state of Wnt receptor complex controls β-catenin degradation or enter into nuclear, thus altering the expression of target genes. Wnt/β-catenin pathway contributes to different types of cancers especially colon cancer [20].

Previous studies revealed that K63-linked ubiquitination of Wnt/β-catenin promote carcinogenesis. Trabid could bind to and deubiquitinate APC, a tumor suppressor protein of Wnt signaling, which regulates β-catenin destruction complex and promotes cancers [21, 22]. In addition, the oncogenic potential of Usp14 has been reported for deubiquitination of Dvl at K444, K451 sties and reinforcement of Wnt signaling of colorectal cancer [23]. It was suggested that E3 ligase Pellino-1 facilitates lung cancer through stabilizing Snail and Slug by K63-linked ubiquitination [24]. Besides, Rad6B mediates K63-linked ubiquitination of β-catenin at K394 site, which regulates the stability and activity of β-catenin in breast cancer [25].

### c-Myc

Highly regulated by various transcriptional regulatory mechanisms, the proto-oncogene c-Myc, participates in multiple growth promoting signaling pathways. Aberrant Myc status impedes genomic stability in relation to tumorigenesis [26].

Several studies suggested that the oncogenic effect of c-Myc is mediated by K63-linked ubiquitination. For example, ZCCHC2 has been reported to suppress RB tumorigenesis via inhibiting HectH9-mediated K63-linked ubiquitination and activation of c-Myc [27]. FBXL6 promotes K63-dependent ubiquitination of HSP90AA1 and its stabilization, which leads to c-Myc activation to promote hepatocellular carcinogenesis [28]. In addition, TRAF6 facilitates hepatocarcinogenesis through interaction with and regulation of HDAC3 K63-linked ubiquitination at K422 to increase c-Myc gene expression and stabilization [29].

### JNK

JNK pathway are responsible for numerous biological processes of protein expression, inflammatory responses, growth, differentiation, survival, and death of cells [30]. JNK functions together with NF-κB, JAK/STAT or other regulatory factors to sustain cell survival. In tumor development, JNK regulates cell apoptosis, cell survival and immune response of cancer [31].

CYLD removes K63-linked ubiquitination c-Fos and c-Jun, and blocks JNK/AP1 signaling, thereby inhibiting tumorigenesis and metastasis of epidermal malignancy [32]. Besides, BLM enhances Fbw7a-mediated c-Jun K63-linked ubiquitylation and suppresses cancer by preventing its oncogenic activity [33].

### YAP/TAZ

As transcriptional coactivators encoded by paralogous genes, YAP and TAZ proteins translocate cytoplasm/nucleus upon different stimulus, including the Hippo pathway [34]. It has been reported that YAP/TAZ remodel cancer cells into cancer stem cells and promote occurrence, growth, and metastasis of cancer [35].

TAK1 has been reported to inhibit YAP/TAZ proteasomal degradation through a complex with E3 ligase TRAF6, thereby promoting their K63-ubiquitination in contrast to K48-ubiquitination [36]. In addition, non-proteolytic K63-linked ubiquitination of YAP controls nuclear translocation and its activity, which is regulated by the E3 ligase complex SKP2 and the deubiquitinase OTUD1 at K321, K497 sites [37].

### Carcinogen

Carcinogen generally includes an agent, mixture, or exposure that lead to carcinogenesis. Carcinogenic chemicals often induce DNA damage to facilitate tumor formation [38].

Carcinogen Cadmium leads to protein aggregates as well as inactivates CYLD to deubiquitinate K63-ubiquitinated proteins and selective autophagy to degrade them [39]. In addition, K63-linked polyubiquitination chains decrease mutagenicity of human lung cells against benzo[a]pyrene-diol-epoxide and contribute to genomic stability [40].

### Others

The K63-linked ubiquitination of other substrates also participate in the regulation of carcinogenesis. For instance, IL-17B/IL-17RB signaling pathway leads to malignant transformation of cancer stem cells via promoting binding of TRAF6 to Beclin-1 and its K63-mediated ubiquitination [41]. Ubiquitination of CALML5 in the nucleus contribute to carcinogenesis of breast cancer, and K63-linked ubiquitination of CALML5 was found in breast cancer tissue, but not in healthy tissue [42]. The E3 ligase HectH9 mediated K63-polyubiquitination of DDX17 upon hypoxia to modulate stem-like and tumor-initiating abilities [43]. Tankyrases bind to and ribosylate LKB1, inducing its K63-linked ubiquitination through RNF146 and inhibiting LKB1 activation. Tankyrase inhibitors activates LKB1, thus promoting AMPK and suppressing tumor [44]. SKP2 regulates Bcr-Abl by inducing its K63-linked ubiquitination and subsequent activation, promoting the initiation and development of chronic myeloid leukemia (CML) [45]. In addition, SKP2 reduces the K63-linked ubiquitination of JARID1B through affecting TRAF6. Inactivating SKP2 inhibits the initiation of prostate cancer via ubiquitination of JARID1B [46]. PLK1 phosphorylates KLF4 and recruits TRAF6, which leads to K63-linked ubiquitination of KLF4 at K32 site and promotes nasopharyngeal cancer [47]. In addition, K95 acetylation of SHMT2 induces SHMT2 degradation by TRIM21-mediated K63 ubiquitination dependent of glucose concentration. Deacetylation of SHMT2 by SIRT3 promotes colorectal carcinogenesis [48].

## K63-linked ubiquitination and tumor growth

One of the most distinct characteristic of cancer cells is unlimited proliferation [49]. Normal cells finely regulate the generation of pro-proliferative signaling pathways, thus keeping stable cell amount as well as maintaining structure and function in tissue. Under the stimulation of multiple carcinogenic factors, the cells escape from normal regulatory mechanism of their growth, and then lead to aberrant cell proliferation [50].

### PI3K/Akt signaling

The activation of Akt is promoted by its K63 ubiquitination, which leads to tumorigenesis. As an E3 ligase, RNF8 activates Akt by K63-linked ubiquitination, which facilitates proliferation of lung cancer cells [51]. In addition, F-box protein FBXL18, the subunit of SCF E3 ligase complex, contributes to glioma growth by facilitating Akt K63-linked ubiquitination [52]. Besides, Skp2-mediated K63-ubiquitination and activation of Akt, which promotes mitochondrial localization of EGF-induced Akt and tumor growth [53]. Stimulation of growth factor promotes dissociation between CYLD and Akt, thus permitting E3 ligases for Akt ubiquitination and activation [54]. BDMC promotes the CYLD expression, mediating Akt K63 deubiquitination and inactivation, which inhibits hepatocellular carcinoma proliferation [55].

### RNF

As a subfamily of ubiquitin ligases, RING finger (RNF) proteins have 49 protein members sharing transmembrane regions. The RING E3 ligases, the major member of RNFs, induce translocation of ubiquitin molecules from E2-ubiquitin intermediates to the substrate [56].

It has been found that RNF8 promotes Twist activation by inducing its K63-linked ubiquitination, which facilitates progression of tumor [57]. Also, RNF8 facilitates c-Myc expression and colon cancer proliferation by mediating β-catenin K63 ubiquitination as well as nuclear translocation [8]. RNF6, another member of RNF family, contributes to proliferation of myeloma cell by inducing K63-linked ubiquitination of glucocorticoid receptor [58]. In addition, RNF181 mediates K63-linked ubiquitination and stabilization of ERα, thus regulating progression of breast cancer [59].

### TRAF

TNFR-associated factors (TRAFs) play crucial role in regulation of IL-17 signaling and proper immune response [60]. TRAFs have been found to function as mediators of multiple stimulation and regulate the downstream activity of many cytokine receptors [61]. TRAFs participate in various biological processes of growth, differentiation, and death of cells [62].

HTLV-1 tax protein regulates MCL-1 stability through TRAF6-mediated K63-linked ubiquitination to promote cell survival [63]. SKP2 knockdown inhibits EZH2 expression prostate cancer cells by promoting TRAF6-induced K63-linked ubiquitination of EZH2 for degradation [64]. Epigallocatechin-3-gallate has been reported to inhibit growth of melanoma cell through suppressing TRAF6 activity [65]. In addition, the link between TRAF6 and autophagy also results in cancer progression. For example, NPM1-mA promotes TRAF6-mediated K63 ubiquitination and stability of ULK1, thus modulating autophagy progress and facilitating proliferation of leukemic cell [66]. TRAF6 interacts with p62 and activates mTORC1 by catalyzing its K63 ubiquitination, which regulate autophagy and cancer cell growth [67]. Autophagy induced by TLR4 or TLR3 activation stimulates multiple cytokine productions via TRAF6 K63-linked ubiquitination and thus facilitates progression of lung cancer cells [68]. Besides, TRAF2 induces K63-linked ubiquitination of DYRK1A, which results in its translocation to vesicles and interaction with SPRY2. Phosphorylated SPRY2 suppresses the endocytosis and recycling of EGFR, which facilitates glioma cell proliferation [69].

### CYLD

As a deubiquitination enzyme (DUB), CYLD could remove the K63-linked polyubiquitin chains from substrates and affect various cellular functions. Decreased CYLD expression is involved in diverse kinds of tumor, CYLD has been regarded as a tumor suppressor gene [70].

CYLD has been reported to interact with and modulate K63-linked ubiquitination of Dvl. Loss of CYLD stimulates tumor growth in human cylindromatosis by hyperubiquitination of Dvl and enhanced Wnt signal [71]. In addition, CYLD phosphorylation impairs its deubiquitinating function, leading to enhanced RIPK1 K63-linked ubiquitination and survival signal of Adult T-cell leukemia/lymphoma cells [72]. Moreover, cancer-related mutations alter CYLD structure and which disturb its binding capacity to K63 ubiquitin molecule. The absence of CYLD DUB activity enhances cancer-promoting function and increases survival of cells [73].

### P53

As a tumor suppressor gene, p53 closely modulates cell proliferation via inducing apoptosis and DNA repair response under stressful conditions [74]. The tumor suppressor p53 exerts multiple functions in the cell by regulating different regulatory signals that ensure accurate cellular responses to stress. p53 status is usually inactive due to ubiquitination by various E3 ubiquitin ligases which target p53 for proteasomal degradation [75].

The K63-linked ubiquitination of p53 has been reported to regulate cancer progression. TRIM31 interacts with p53 and mediates its K63-linked ubiquitination to inhibit breast cancer development [76]. TRIM45 suppresses tumor progression in the brain by stabilizing p53 through K63-linked ubiquitination [77]. TRAF6 restricts amount of p53 in mitochondria by inducing K63-linked ubiquitination of p53 at K24 site in cytoplasm. In addition, TRAF6 facilitates the K63-linked ubiquitination of nuclear p53, which therefore influence apoptosis and tumor inhibition [78].

### Cell cycle

Cell cycle is a complicated process that involves numerous regulatory proteins that dictates the cell through a series of events culminating in mitosis and the generation of two new cells [79]. Cell cycle process is orchestrated by sequential activation of cyclin-dependent kinases (CDKs) by their corresponding cyclin partner. The cell cycle represents an irreversible process that sustains multiple sequenced events controlled by three key checkpoints [80].

DZIP3 interacts with and promotes K63-linked ubiquitination and stabilization of Cyclin D1, which drives cell cycle and cancer progression [81]. In addition, ERLIN2 induces K63-linked ubiquitination of Cyclin B1 to stabilize it for modulating cell cycle progression of breast cancer [82]. Besides, FBW7 suppresses cell growth and G2/M cell cycle transition by inducing K63-linked ubiquitination of γ-catenin [83].

### TRIM

Tripartite motif (TRIM) family proteins, most of which possess E3 ubiquitin ligase activities because they contain a RING-finger domain, play various roles in cellular processes including intracellular signaling, autophagy, apoptosis, protein quality regulation, innate immunity, development, and carcinogenesis [84].

TRIM9s induces the K63-linked ubiquitination of MKK6 at K82, thus suppressing the K48-linked ubiquitination of MKK6 at this site responsible for degradation. In turn, MKK6 increases stability of TRIM9s by inducing its phosphorylation by p38, thus inhibiting its ubiquitin-proteasome degradation. The interaction and stabilization of TRIM9s with MKK6 enhance p38 pathway to suppress progression of glioblastoma [85]. Besides, TRIM56 keeps stability of ER alpha protein by targeting its K63-linked ubiquitination to enhance estrogen signaling and growth of breast cancer [86].

### Metabolism

Metabolism refers to various kinds of reactions in organisms that can maintain the process of life. Metabolic processes involve multiple interrelated cellular pathways that ultimately provide cells with the energy they need to perform their functions [87]. It has long been known that cancers have remodeled metabolism pattern to help meet the needs of cells that have the potential for uncontrolled proliferation.

The ubiquitin ligase HectH9 hijacks Hexokinase 2 (HK2) to mitochondria K63-linked ubiquitination for promoting its dual functions in glycolysis and apoptosis suppression, which in turn contribute to tumor development [88]. Furthermore, PSMD14 suppresses K63-linked ubiquitination of PKM2 and pyruvate kinase activity, which promotes its nuclear translocation and leads to aerobic glycolysis and progression of ovarian cancer [89].

### Others

Some other regulation of K63-linked ubiquitination also participates in the growth of tumor. As an E2 enzyme specific for K63-linked ubiquitin, UBE2N has been found to potentiate melanoma proliferation through MEK-FRA1-SOX10 pathway [90]. USP10 removes TRIM25-induced K63 polyubiquitination of PTEN and activate it, thus inhibiting growth of lung cancer [91]. Ubiquitin E3 Ligase Pellino-1 suppresses IL-10-mediated polarization of macrophage by IRAK1 K63 ubiquitination and STAT1 activation, which leads to decreased tumor proliferation rate [92]. Skp2-SCF induces K63-linked ubiquitination of LKB1, which regulates its activation and liver cancer growth [93]. USP1 has been found to deubiquinate K63-linked ubiquitination of ULK1, which modulates autophagy and tumor growth [94]. GASC1 transcriptionally represses ubiquitin ligase FBXO42, thus reducing degradation of ROCK2 via K63 ubiquitination and promoting growth of hepatocellular carcinoma [95]. NEDD4 ubiquitin E3 ligase catalyzes K63-linked ubiquitination of IGPR-1, resulting in its lysosomal-dependent degradation to suppress angiogenesis and tumor growth [96]. Skp2 induces K63-linked ubiquitination of MTH1, which promotes its stability and growth of melanoma cells upon oxidative stress [97]. Moreover, hypoxia induces K63 ubiquitination of HAUSP and subsequent HIF-1α deubiquitination, which induces H3K56 acetylation by CBP on promoters of HIF-1α target genes [98].

## K63-linked ubiquitination and tumor invasion

The invasive behavior of cells is a unique sign of cancer, which is characterized by the invasion of cells to change the original cellular environment. The steps of cell invasion include cell adhesion, degradation of extracellular matrix proteins and final cell migration [99]. Cancer development is characterized by the capacity of cancer cells to exploit an innate migratory ability to invade peri-tumor tissues [100].

### Breast cancer

Breast cancer, the most frequently occurred cancer and the leading cause of cancer-related death of women, is affected by multiple genetic and epigenetic factors. Breast cancer is classified as hormone receptor positive, HER2 positive and triple-negative breast cancer on the basis of certain characteristics [101].

Several studies unraveled the critical of K63-linked ubiquitination in regulating breast cancer invasion. For example, Ubc13-Uev1A complex activates AKT pathway via K63-linked ubiquitination and increases CT45A expression, resulting in cell migration and EMT of breast cancer cells [102]. TRIM59 suppresses K63 ubiquitination by RNFT1 and PDCD10 degradation by p62-mediated selective autophagy, which promotes migration of breast cancer cells [103]. Besides, K63-linked ubiquitination was suggested as a regulator of arachidonic acid-induced adhesion and migration of cells [104].

### Lung cancer

Lung cancer is a malignant tumor with the high incidence rate and mortality in the world. Emerging epidemic studies reveal that smoking, air pollution, harmful occupational exposure, genetic susceptibility, radiation exposure are responsible for high incidence of lung cancer [105]. Lung cancer are divided into small cell carcinoma and non-small cell carcinoma, which are useful for prognosis evaluation and therapy decisions [106].

TRAF6 upregulation and K63-linked ubiquitination is found in lung cancer cells, while TRAF6 knockdown suppresses the invasion of lung cancer cells [107]. TRIM37 promotes K63-linked ubiquitination of TRAF2, activating the NF-κB pathway and enhancing the aggressive behaviors of NSCLC cells [108]. In addition, K63-linked ubiquitination of TRAF4 promotes aggressiveness of lung cancer by remodeling tumor microenvironment of certain fibroblasts [109].

### Nervous system cancer

Gliomas are tumors of central nervous system that originate from oligodendrocytes or astrocytes [110]. As a common and malignant brain cancer, the morphological characteristics of glioblastoma are excessive cell structures and mitotic behavior, necrosis and vascular proliferation [111]. Neuroblastoma is an embryonic tumor that occurs in the tissues of the sympathetic nervous system [112].

E3 ubiquitin ligase Nedd4-1 induces K63 ubiquitination of Rap2a and promotes the migration as well as invasion of glioma cells [113]. In addition, CaMKII phosphorylates Beclin 1 to promote its K63 ubiquitination and subsequent activation of autophagy, which contributing to the differentiation of neuroblastoma cells [114]. Moreover, Nrdp1 binds to the Vangl1 and Vangl2 proteins to mediate K63 ubiquitination of the wnt pathway protein Dishevelled (Dvl), regulating the invasiveness and malignancy of glioblastoma [115].

### Gastric cancer

Gastric cancer is a common digestive tract tumor with high incidence rate and mortality [116]. Carcinogenesis of gastric cancer is a multifactorial process regulated by microbial, environmental, and genetic factors, although *Helicobacter pylori* infection is regarded as the primary cause [117].

CPT1A succinylates LDHA at K222 and impairs the interaction of K63-ubiquitinated LDHA with p62, which inhibiting LDHA degradation and potentiating invasion of gastric cancer cells [118].

## K63-linked ubiquitination and tumor metastasis

Migration represents the cellular movement across the tissue, which includes migration of single cell and grouped cells [99]. Metastatic behavior is the final outcome of the multi-step cell process of invasion and metastasis, which means that cancer cells spread far away and adapt to the tissue microenvironment of different locations [119].

### Breast cancer

TRAF6/USP17 regulates the K63-linked ubiquitination of AEP, and cooperates with HSP90α to facilitate metastasis of breast cancer cells [120]. Ubiquitin-conjugating enzyme Ubc13, an E2 enzyme responsible for K63-linked protein ubiquitination, promotes metastasis of breast cancer through a TAK1-p38 MAP pathway [121]. Suppression of TRIM59, a highly expressed E3 ligase in breast cancer with metastasis, inhibits metastasis by inducing RNFT1-induced K63 ubiquitination of PDCD10 [122]. Besides, OTUD7B deubiquitinates LSD1 at K226/277 sites, leading to dynamic binding regulation of LSD1 and further metastasis of breast cancer cells [123].

### Digestive cancer

Digestive system cancers are the most common malignancies, which mainly include gastric cancer, esophageal cancer, colorectal cancer, liver cancer, and pancreatic cancer [124]. Colorectal cancer is one of the most frequent neoplasms, most of which are localized with or without lymph node metastases [125]. Hepatocellular carcinoma is a common malignancy with an increasing worldwide prevalence, which usually develop on the basis of chronic liver disease [126].

Uev1A-Ubc13 promotes K63-linked ubiquitination of CXCL1 expression and NF-кB activation, thus regulating metastasis of colorectal cancer [127]. TRAF6 binds to MAP1LC3B/LC3B and induces LC3B K63-linked ubiquitination, which inhibiting colorectal cancer metastasis via regulating degradation of β-catenin by selective autophagy [128]. In addition, Trabid cooperates with Twist1 to specifically removes RNF8-mediated K63 ubiquitin chains from Twist1, thus suppressing hepatocellular carcinoma metastasis [129].

### Others

RNF8 mediates K63-linked polyubiquitin and stabilization of Slug, promoting Epithelial-Mesenchymal Transition of lung cancer cells [130]. In addition, TRAF6 modulates invasion as well as metastasis of melanoma via Basigin ubiquitination and BSG-dependent MMP9 induction [131].

## K63-linked ubiquitination and apoptosis

Known as a highly conserved programmed cell death, apoptosis is a rational and active decision made to sacrifice certain cells for the better benefits of the organism [132]. Apoptosis plays key roles in various cellular processes, including homeostasis, development, immunity cell survival and fate [133].

### TNF/TNFR

Tumor necrosis factor is an essential cytokine responsible for signaling pathway of immune response [134]. TNF-alpha, TNF-beta represent the most important TNF members. TNF receptors induce two distinct pathways: TNFR1 participates in apoptosis pathway. In contrary, TNFR2 is involved in cell survival pathways [135].

USP4 potentiates TNF-α-mediated apoptosis by deubiquitinating RIP1 in head and neck squamous cell carcinoma [136]. RACK1 recruits the E3 ligase TRAF2 to MOAP-1 to facilitate K63 ubiquitination, which interacts with Bax for apoptosis [137]. ASK1-induced phosphorylation of Daxx promotes K63-linked ubiquitination of Daxx at Lys122, which further increases ASK1 activity by a positive feedback loop and regulate apoptosis [138]. Moreover, knockdown of miR-182 upregulates CYLD and RIP1 deubiquitination, which activates caspase-8 and apoptosis in triple-negative breast cancer cells [139].

### EBV

As one of the most widespread human virus, Epstein-Barr virus (EBV) infection causes life-long latent infection, which leads to various tumorigenic diseases [140]. The interaction of EBV latent genes with oncogenes contribute to aberrant cell cycle, thereby promoting the development of EBV-associated neoplasms [141].

Upon proteasomal block, K63-linked ubiquitination of EBNA3C is induced for co-localization with certain autophagy-lysosomal components of the cytoplasm, which induces cell death in B-lymphocytes through apoptosis [142]. In addition, LMP1 promotes K63 ubiquitination of p53 via TRAF2, thereby contributing to p53 accumulation and disrupting p53-induced apoptosis [143].

### Mitochondria

Mitochondria are well known for its function of ATP production by oxidative phosphorylation [144]. In addition, production of lipids and amino acids, degradation of fatty acids, the urea cycle also occur within mitochondria [145]. Mitochondria supports cell function and determinates cell death pathways, which is involved in aberrant metabolism and tumorigenesis [146].

Vorinostat and quinacrine increase intracellular ROS and promote the accumulation of K63-linked ubiquitination of the mitochondria, leading to mitochondrial dysfunction and apoptosis in T-cell acute lymphoblastic leukemia [147]. Besides, K63-linked ubiquitination of Hexokinase 2 induced by HectH9 modulates its mitochondrial localization and function, which controls tumor metabolism and apoptosis [148].

### Others

TRIM25 induces K63-linked ubiquitination of PTEN at K266, which activates the AKT/mTOR pathway and regulates NSCLC growth and apoptosis under stimulation [149]. USP9X interacts with FLT3-ITD and induces its K63-linked ubiquitination while FLT3-ITD promotes degradation of USP9X via the ubiquitin-proteasome pattern, the cooperation of which controls apoptosis in AML cells [150]. Small-molecule WP1130 selectively blocks DUB activity of USP14, USP5, UCH37 and USP9x, decreasing antiapoptotic and increasing proapoptotic proteins, such as MCL-1 and p53 [151]. Selenite increases CYLD by downregulating LEF1 and cIAP, both of which lead to deubiquitination of RIP1 and apoptosis of colorectal cancer cells [152]. WP1130 blocks kinase signaling by inhibiting Usp9x deubiquitinase activity and Bcr-Abl ubiquitination, thus inducing apoptosis CML cell [153]. In addition, E3 ubiquitin ligase HECTD3 was suggested to interact with and induce K63-linked ubiquitination of caspase-8 which impair its activation [154].

## K63-linked ubiquitination and immune

Immunity is a physiological function that destroy and reject antigenic substances that enter the body, or damage cells and tumor cells produced by the body itself, in order to maintain the health of the human body [155]. The immune system consists of intrinsic immunity and adaptive immunity, which is further divided into humoral and cellular immunity, the disregulation of which contribute to caicinogenesis [156].

### T cell

T cells regulate all aspects of adaptive immunity throughout life, including responses to pathogens, allergens, and tumors. T cells are indispensable for the establishment and maintenance of immune response, homeostasis, and memory [157]. Receptors expressed by T cells are responsible for recognizing a variety of antigens from pathogens, tumors, and the environment, and maintaining immune memory and tolerance [158].

Regulatory T cells (Tregs) are key regulators of immune control, the suppression of which largely rely on FOXP3 transcriptional activity. E3 ligase TRAF6 mediates K63-linked ubiquitination of FOXP3 at K262, which ensures its proper localization and subsequent functions of Tregs [159]. In addition, CD137 promotes NF-κB activation in a K63-linked ubiquitination-dependent manner mediated by TRAF2, and CD137 antibodies potentiate CD8-related anti-tumor immune response [160].

### Th9

Th9 cells, a specific helper T cell subset that specifically secretes cytokine IL-9, might be involved in host reaction towards pathogen, immune response to tumor, and immune-related disorders, such as allergic and autoimmune diseases [161, 162].

BFAR induces K63-linked ubiquitination on TGFβR1 at K268 site, which mediates TGFβ signaling activation and Th9-mediated cancer immunotherapy [163]. In addition, transcription factor PU.1 selective degradation via K63 ubiquitination in autophagy inhibits the differentiation and anti-tumor ability of Th9 cells [164].

### MDSC

Myelogenous suppressor cells (MDSC) are a special subgroup of immunosuppressive myeloid cells. Their accumulation under chronic inflammatory conditions is one of the important characteristics of cancer [165]. MDSC significantly limits the antitumor activity of T and NK cells, and mediates the aggregation along with initiation of immunosuppressive cells such as Treg and M2 macrophages, thereby promoting tumor progression [166].

TRAF6 promotes the immunosuppression of MDSCs via inducing K63-linked ubiquitination and phosphorylation of STAT3, which might become a potential target for antitumor immunotherapy [167]. Besides, silencing p66a leads to phosphorylation as well as K63 ubiquitination of STAT3, thus promoting accumulation, differentiation, and activation of MDSC [168].

### Others

*Helicobacter pylori* virulent factor CagA interacts with SHP-1 and target TRAF6 for K63-Linked ubiquitination, thereby inhibiting the expression of proinflammatory cytokines and subsequent immune response [169]. DAPK3 phosphorylates the E3 ligase LMO7 at S863, resulting in LMO7-STING interaction, K63-linked polyubiquitination of STING, and tumor-intrinsic immunity [170]. USP22 removed K63-linked ubiquitination of PD-L1 as well as CSN5, which regulating PD-L1 abundance via USP22/CSN5/PD-L1 signal. USP22 knockout suppresses tumorigenesis and increases the cytotoxicity of T cell [171]. Furthermore, HER2 recruits AKT1 to directly phosphorylate TBK1, which impairs the TBK1-STING association and K63-linked ubiquitination of TBK1, thus disrupting STING signaling and inhibiting antitumor immunity [172].

## K63-linked ubiquitination and NF-κB Signaling

The nuclear factor kappa B (NF-κB) transcription factors family mainly regulate inflammation response, immunity, and tumor [173]. Aberrant NF-κB signaling has been reported to participate in multiple diseases of inflammatory or immune disorders and cancer [174].

### TRAF2/5

The tumor necrosis factor receptor-associated factor (TRAF) family members are adaptor proteins regulating inflammation, adhesion, growth, differentiation, and apoptosis [175]. TRAF2 is a prototypical TRAF member, which associates with canonical as well as non-canonical NF-κB signal [176]. Another important member TRAF5 could interact with LTβR to activate NF-κB [175].

Several studies indicated the involvement of TRAF2 in regulating NF-κB activation. Siva-1 suppresses NF-κB activation by K63-ubiquitination of TRAF2, thus regulating homeostasis and memory of T-cell [177]. TRIM31 increases nuclear p65 by mediating K63-linked ubiquitination of TRAF2 and NF-κB activation of pancreatic cancer [178]. E3 ligase complex cIAP1/cIAP2/TRAF2 triggers IKKε K63-linked polyubiquitination, which is critical for NF-κB activation and malignant transformation breast cancer cells [179]. In addition, GOLPH3 promotes K63-linked ubiquitination of RIP, NEMO and TRAF2, which causes NF-κB activation and aggressiveness of hepatocellular carcinoma cells [180]. Besides, E2 regulatory protein of α, β and µ-HPV genotypes promotes TNF-induced NF-κB activation via K63-linked ubiquitination mediated TRAF5 activation [181].

### IKKβ/IKK

Blocking nuclear factor-κB (IκB) kinase (IKK) complex mainly regulate the NF-κB signaling pathway [182]. IKKalpha and IKKbeta mediate IkappaB phosphorylation, of which IKKbeta are responsible for rapid NF-κB activation by proinflammatory signaling pathways while IKKalpha activates a certain forms of NF-κB in reaction to a portion of TNFs [183].

The pVHL-mediated K63-ubiquitination of IKKβ, a key modulator of NF-κB, impairs TAK1 binding, which inhibits IKKβ phosphorylation and activation of NF-κB [184]. cIAP1/2 mediates K63-linked ubiquitination of themselves as well as BCL10, recruiting and activating IKK [185]. Inhibiting UBC13-UEV1A complex controlling K63-linked ubiquitination suggests K147 as the main site of K63 ubiquitination and necessary for activation of STAT3 [186]. Furthermore, K63-linked ubiquitination occurs in K578 in BRAF V600E other homologous to IKKβ K147, which drives melanoma and other cancers [187].

### RIP1

The receptor-interacting protein (RIP1) is widely expressed and belongs to a kinase family which induces responses to stress or inflammation of cells, thus determining cell survival or death [188]. RIP1 is closely implicated in apoptosis-related cellular death induced by TNFα stimulation as well as in necrotic pattern of cell death induced when caspase is inactivated [189].

Silencing miR-138 induces K63-linked ubiquitination of RIP1 and sustains activation of NF-κB as well as esophageal squamous cell carcinoma progression [190]. MicroRNAs miR-125a and miR-125b target TNFAIP3, which altering K63 ubiquitination of RIP1 and transcription of NF-κB target genes [191]. EGFRvIII mediates RIP1 K63 ubiquitination while RIP1 interacts with NEMO and TAK1 to activate NF-κB, which modulates tumorigenesis and efficacy of targeted treatment in GBM [192]. FLOT1 promotes tumor necrosis factor-α receptor signaling via mediating its K63-linked ubiquitination and activates NF-κB in ESCC cells [193]. In addition, p62 has been reported as an oncotarget regulates cisplatin sensitivity of human ovarian cancer cells via activating RIP1-NF-κB pathway in a K63-linked ubiquitination manner [194].

### CYLD

The HPV-encoded E6 protein mediates activation of NF-κB under hypoxia by targeting CYLD K63 deubiquitinase which negatively regulate NF-κB pathway [195]. In addition, CYLD protein with D681G mutation could not cleave K63-linked polyubiquitin chains, significantly impairing its capacity to inhibit TRAF2- and TRAF6-induced NF-κB activation and to deubiquitinate TRAF2 [196].

### Others

TRIM14 decreases K63 ubiquitination of p100/p52 by recruiting deubiquitinase USP14, thus inhibiting selective autophagic degradation of p100/p52 induced by p62 and promoting noncanonical activation of NF-κB [197]. Triggering of MSR1 mediated through K63 polyubiquitylation in macrophages with activated IL-4 promotes JNK signal, thus changing from anti-inflammation into pro-inflammation [198]. TRIM22 contributes to NF-κB activation by binding with IKKγ and facilitating its K63-linked ubiquitination, which results in phosphorylation of IKKα/β and IκBα in glioblastoma [199]. Moreover, RNF138 promotes K63 ubiquitination of MYD88L265P, thus recruiting of kinases in relation with interleukin-1 receptor and activating NF-κB in lymphomas [200].

## K63-linked ubiquitination and DNA damage repair

DNA repair system protect cells from the endogenous and exogenous insults, which prevents tumorigenesis [201]. DNA repair systems maintain genetic integrity and stability, including base excision repair (BER), mismatch repair (MMR), nucleotide excision repair (NER) and double-strand break repair (DSBR) [202].

### DSBR

DNA double-strand breaks (DSBs) are harmful lesions that lead to genetic insults. To avoid genome instability, several DSBR pathways exist in organisms including non-homologous end-joining (NHEJ) and homologous recombination (HR) as the two most commonly adopted systems [203].

#### NHEJ

NHEJ is known as an error-prone pattern and independent of homologous DNA for end joining [204].

USP38 decreases the K63-linked ubiquitination of HDAC1 and promote its deacetylase activity, thus deacetylating H3K56. USP38 deletion reduces NHEJ efficiency and increases genome instability, which potentiates sensitivity of cancer cells to genotoxic insults [205]. Moreover, FBXW7 has been found to facilitate NHEJ via K63-Linked ubiquitylation of XRCC4 at lysine 296, thus interacting with the Ku70/80 complex to promote NHEJ repair [206].

#### HR

HR is largely error free which needs extensive homology for repairing DNA DSBs [207].

K63-linked ubiquitination of RYBP keep it away from DNA damage sites, which impairs BRCA1 recruitment and Rad51 to DNA double-strand breaks, thus suppressing HR repair. As a result, cancer cells with high RYBP expression are more sensitive to DNA damage therapy [208]. Skp2 E3 ligase interacts with NBS1 and promotes its K63-linked ubiquitination in response to DSBs, which is important for NBS1-ATM interaction, thus recruiting ATM to the DNA foci for further activation [209]. In addition, FANCG K63 ubiquitination mediates its interaction with the Rap80-BRCA1 complex for the regulation of HR repair [210].

#### Other DSBR

E3 ubiquitin ligase ITCH could trigger K63-linked ubiquitination of WWOX at K274 site and regulate the nuclear accumulation of WWOX, which is critical for ATM activation and DNA repair [211]. SOCS1 leads to nuclear redistribution and K63 ubiquitination of VHL under DSBs, while VHL loss impairs the DDR [212]. Tax promotes RNF8 for nuclear K63-linked ubiquitination which sequester DDR factors of Tax speckles, inhibiting DDR as well as DSB repair in Adult T-Cell Leukemia cells [213]. USP19 cleaves K63-linked ubiquitin of HDAC1/2, which modulates HDAC1/2 activity upon DNA damage repair [214]. Moreover, it has been found that the interaction of K63-linked ubiquitin molecules with DNA recruits repair effector via their interaction with an Ile patch in ubiquitin to promote DNA repair upon DNA damage [215].

### Others

Deubiquitinase CYLD promotes DNA damage-induced p53 activation by removing K48-ubiquitin chains from p53 and cleaving K63-ubiquitin, which regulates p53 responses to genotoxic stress in cancer cells [216]. Furthermore, UBC13 mediates K63-linked PCNA ubiquitination, which regulates DNA damage-induced replication fork slowing and reversal during eukaryotic replication [217].

## K63-linked ubiquitination and cancer therapy

Tumor therapy mainly includes surgery, chemotherapy, radiotherapy, biotherapy and molecular targeted therapy. Generally, surgery is the main treatment for most tumors, but some patients need chemotherapy, radiotherapy and other treatments [218].

### Chemotherapeutic drug

Chemotherapy refers to the treatment that uses chemical agents to kill cancer cells, inhibit the growth of tumor cells or promote their differentiation. According to the specific mechanism, commonly used chemotherapeutic agents can be classified into different classes [219].

Nuclear XIAP increases NFκB expression and K63-ubiquitination, which influences drug resistance and confers poor prognosis in breast cancer [220]. IRAK1/4 signaling promotes activation of the E3 ubiquitin ligase TRAF6, which triggers K63-linked ubiquitination and stabilization of antiapoptotic protein MCL1, thus decreasing sensitivity of T-ALL to combined therapy [221]. SMO stabilizes TRAF6 via recruiting USP8 to remove its K48 ubiquitination, which is associated with enhancement of TRAF6 K63 ubiquitination, thereby regulating AKT activation and cause doxorubicin resistance in diffuse large B cell lymphoma [222]. Moreover, SphK2 promotes the RXRα degradation dependent of K63-linked ubiquitination in autophagy, resulting all-trans retinoic acid (ATRA) therapy insensitivity of colon cancer [223].

### Targeted drug

The classic commonly used chemotherapy drugs generally act directly on the DNA of cells, while new anticancer drugs include molecular targeted therapy, such as targeting the abnormally expressed indicators in cancer cells [224]. Targeting drugs usually function in several ways including enzyme mediation, pH-dependent delivery, special vehicles transport and receptor targeting [225].

Ubiquitin ligase TRIM15 and deubiquitinase CYLD regulate K63-linked ubiquitination of ERK and its interaction with MEK and subsequent activation. Decrease of TRIM15 suppresses proliferation of melanomas, which might become potential target for cancer therapy [9]. RBX1 activates POLR2A which encodes RNAP2 catalytic subunit through K63 ubiquitination and increases the RNAP2-induced biosynthesis of mRNA. Synergistic suppression of RBX1 and RNAP2 inhibits prostate cancer development, which promotes the therapeutic sensitivity of the RNAP2 inhibitor [226]. ErbB2/HER2 receptor tyrosine kinase is a validated clinical target for increasing number of anti-ErbB2 therapeutics. E3 ubiquitin ligase c-Cbl and deubiquitinase USP9x regulate ErbB2 trafficking as well as degradation in relation to K48 or K63 ubiquitination [227]. Recombinant monoclonal antibody Trastuzumab targets ErbB family members against cancer. ATG9A loss has been found to confer resistance to trastuzumab through c-Cbl induced Her2 K63 ubiquitination [228]. In addition, TRIM32 is responsible for K63-linked ubiquitination and activation of TBK1 upon EGFR suppression, which exerts efficacy in treating non-small cell lung cancer [229].

## Summary and future directions

**Fig. 1:**
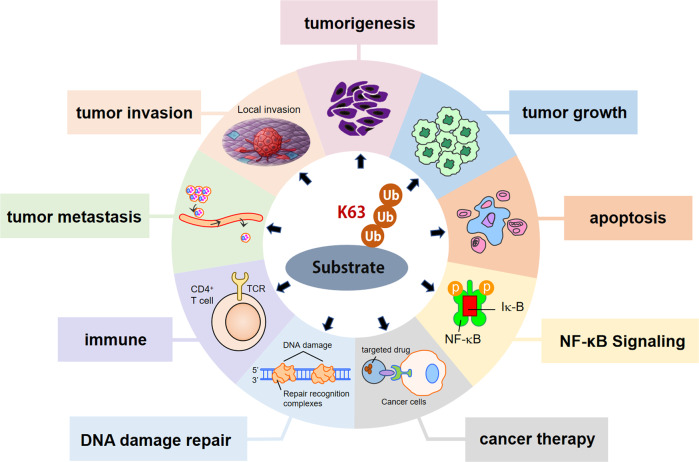
K63-linked ubiquitination regulates multiple aspects of cancer. Various aspects of tumor have been reported to beclosely regulated by K63-linked ubiquitination including tumorigenesis, tumor growth, tumor invasion, tumormetastasis, immune, apoptosis, NF-κB signaling, DNA damage repair and cancer therapy.

As a specific pattern of post-translational modifications, K63-linked ubiquitination controls various properties of protein including protein-protein interaction, translocation, and activation. Emerging evidence indicates that K63-linked ubiquitination participates in the initiation, development, and therapy of cancer. However, there was previously a lack of an overview of the contribution of K63-linked ubiquitination in different aspects of tumor. Therefore, our review summarized recent advances of studies focusing on the critical implication of K63-linked ubiquitination in cancer (Fig. 1) (Supplementary Table 1).

Tumorigenesis, tumor growth, invasion, and metastasis are complicated processes regulated by multiple pathways such as PI3K/Akt signaling, c-myc, JNK, YAP, p53 and Wnt/β-catenin. Previous studies suggested that various factors of these pathways are regulated by K63-linked ubiquitination. As for different cancer types, K63-linked ubiquitination participate in the regulation of numerous cancers including breast cancer, gastric cancer, colorectal cancer, hepatocellular carcinoma, which indicates most cancers are modulated by this specific ubiquitination pattern. In addition, diverse cellular processes including cell cycle, DNA damage repair, NF-κB signaling, autophagy and mitochondria function require proper K63-linked ubiquitination of certain members. Furthermore, exciting discoveries are anticipated to unravel switches of different Ub chains (such as between K48 and K63) in cancer.

Chemotherapy refers to the treatment that uses chemical agents to kill cancer cells, while targeting drugs usually function by special vehicles transport and receptor targeting. In addition, anti-tumor immune turn out to be a promising approach to kill tumor cells. Extensive studies reported that K63-linked ubiquitination regulate multiple cancer therapies of chemotherapy, target drug, and anti-tumor immune, which offer a novel way for cancer treatment by targeting distinct aspects of the ubiquitin system (Supplementary Table 2). Multiple E3 ligases of TRAFs, RNFs, TRIMs, and DUBs of USPs, CYLD, OTUDs participate in the modulation of K63-linked ubiquitination of different substrates. Development of novel therapeutic approaches could be promising that selectively target interaction of proteins, thus altering the binding of various Ub to conjugation enzymes or Ub receptors. Small molecules targeting certain E3 ligase or DUBs might exert favorable effect to suppress tumor development and metastasis. Along with increasing technologies and comprehensive understanding of ubiquitination signal, it is anticipated that novel therapeutic strategies improve tumor therapy.

## Supplementary information

Supplementary Tables

## Data Availability

All the data used in the manuscript are freely available online.
