# Supplementary material for: Role of K63-linked ubiquitination in cancer
Source: Cell Death Discov. 2022 Oct 6;8:410. doi: 10.1038/s41420-022-01204-0 (PMC9537175; doi:10.1038/s41420-022-01204-0)
Supplement: Supplementary file 1 — Supplementary Tables [file 41420_2022_1204_MOESM1_ESM.docx]

**Supplementary Table 1. Characteristics of the studies investigating the association of K63-linked ubiquitination with cancer.**

| **Aspects** | **Author** | **Year** | **E3** | **DUB** | **Substrate** | **Site** | **Function** |
| --- | --- | --- | --- | --- | --- | --- | --- |
| 1.1 PI3K/Akt signaling |  |  |  |  |  |  |  |
|  | Han, F. | 2018 | Skp2 SCF complex |  | Akt |  | Skp2 SCF complex mediates the K63-linked ubiquitination and activation of Akt, which is responsible for subsequent oncogenesis |
|  | Wang, G. | 2019 | SETDB1 |  | Akt |  | SETDB1-mediated methylation of Akt promotes its K63-linked ubiquitination and activation leading to tumorigenesis |
|  | Wang, B. | 2017 | TRAF2 | OTUD7B | Akt |  | E3 ubiquitin ligase TRAF2 and deubiquitinating enzyme OTUD7B control a K63-linked polyubiquitination switch of GβL that modulates activation of mTORC2/AKT signalling |
| 1.2 Wnt/β-catenin |  |  |  |  |  |  |  |
|  | Gerard, B. | 2012 | Rad6B |  | β-catenin | K394 | Rad6B mediates K63-linked ubiquitination of β-catenin, which regulates the stability and activity of β-catenin in breast cancer |
|  | Jeon, Y. K. | 2017 | Pellino-1 |  | Slug and Snail |  | E3 ligase Pellino-1 promotes lung carcinogenesis through the stabilization of Slug and Snail by K63-linked ubiquitination |
|  | Jung, H. | 2013 |  | Usp14 | Dvl | K444, K451 | USP14-mediated K63-linked deubiquitination of Dvl exerts an oncogenic role via enhancement of Wnt/β-catenin signaling |
|  | Tran, H. | 2008 |  | Trabid | APC |  | Trabid can bind to and deubiquitylate the APC tumor suppressor protein, which promotes cancers |
|  | Tran, H. | 2012 |  | Trabid | APC |  | Trabid can mediate K63-linked ubiquitination of the APC tumor suppressor protein to regulate β-catenin destruction complex |
| 1.3 c-MYC |  |  |  |  |  |  |  |
|  | Dai, H. | 2020 | HectH9 |  | c-Myc |  | ZCCHC2 suppress RB tumorigenesis via inhibiting HectH9-mediated K63-linked ubiquitination and activation of c-Myc |
|  | Shi, W. | 2020 | FBXL6 |  | HSP90AA1 |  | FBXL6 promoted K63-dependent ubiquitination of HSP90AA1 and its stabilization, which leads to c-Myc activation to promote hepatocellular carcinogenesis |
|  | Wu, H. | 2020 | TRAF6 |  | HDAC3 | K422 | TRAF6 facilitates hepatocarcinogenesis through regulation of HDAC3 K63-linked ubiquitination to increase c-Myc gene expression and stabilization |
| 1.4 JNK |  |  |  |  |  |  |  |
|  | Miliani de Marval, P. | 2011 |  | CYLD | c-Jun and c-Fos |  | CYLD deubiquitinates K63-linked of c-Fos and c-Jun and blocks JNK/AP1 signaling to inhibit tumorigenesis and metastasis of epidermal malignancy |
|  | Priyadarshini, R. | 2018 | Fbw7α |  | c-Jun |  | BLM enhances Fbw7a-mediated K63-linked ubiquitylation of c-Jun and function as a tumor suppressor by preventing its oncogenic activity |
| 1.5 YAP/TAZ |  |  |  |  |  |  |  |
|  | Santoro, R. | 2020 | TRAF6 |  | YAP/TAZ |  | TAK1 inhibits YAP/TAZ proteasomal degradation through a complex with E3 ligase TRAF6, thereby promoting their K63-ubiquitination |
|  | Yao, F. | 2018 | SKP2 | OTUD1 | YAP | K321, K497 | The SCF(SKP2) E3 ligase complex and the deubiquitinase OTUD1 regulate non-proteolytic K63-linked ubiquitination of YAP, which controls its nuclear translocation and activity |
| 1.6 caicinogen |  |  |  |  |  |  |  |
|  | Chargui, A. | 2021 |  | CYLD |  |  | Carcinogen Cadmium induce the formation of protein aggregates that sequester and inactivate CYLD and selective autophagy, that deubiquitinate and degrade K63-ubiquitinated proteins respectively |
|  | Langie, S. A. | 2007 |  |  | PCNA |  | K63-linked poly-ubiquitin chains protects human lung cells against benzo[a]pyrene-diol-epoxide-induced mutagenicity and contribute to genomic stability |
| 1.7 Others |  |  |  |  |  |  |  |
|  | Bie, Q. | 2021 | TRAF6 |  | Beclin-1 |  | IL-17B/IL-17RB signaling pathway leads to self-renewal and tumorigenesis of cancer stem cells by promoting binding of TRAF6 to Beclin-1 and its K63-mediated ubiquitination |
|  | Debald, M. | 2013 |  |  | CALML5 |  | K63-linked ubiquitination of CALML5 in the nucleus contribute to carcinogenesis of breast cancer |
|  | Kao, S. H. | 2019 | HectH9 |  | DDX17 |  | The E3 ligase HectH9 mediated K63-polyubiquitination of DDX17 upon hypoxia to modulate stem-like and tumor-initiating abilities |
|  | Li, N. | 2019 | RNF146 |  | LKB1 |  | Tankyrases promote RNF146-mediated LKB1 K63-linked ubiquitination and induce tumorigenesis |
|  | Liao, Y. | 2019 | SKP2 |  | Bcr-Abl |  | SKP2 mediate Bcr-Abl K63-linked ubiquitination and activation, promoting development and progression of CML |
|  | Lu, W. | 2015 | TRAF6 |  | JARID1B | K242 | Skp2 decreased the K63-linked ubiquitination of JARID1B by TRAF6, promoting prostate tumorigenesis |
|  | Mai, J. | 2019 | TRAF6 |  | KLF4 | K32 | PLK1-TRAF6-KLF4 feed-forward loop leads to tumorigenesis in NPC |
|  | Wei, Z. | 2018 | TRIM21 |  | SHMT2 |  |  |
| 2.1 PI3K/Akt signaling |  |  |  |  |  |  |  |
|  | Qiu, C. | 2020 |  | CYLD | Akt |  | BDMC inhibits hepatocellular carcinoma proliferation through Akt inactivation via CYLD-mediated deubiquitination |
|  | Xu, Y. | 2021 | RNF8 |  | Akt |  | RNF8-mediated regulation of Akt promotes lung cancer cell survival and resistance to DNA damage |
|  | Yang, W. L. | 2013 |  | CYLD | Akt |  | CYLD, a molecular switch for the cycles of ubiquitination and deubiquitination, regulates growth factor-mediated activation of Akt signaling |
|  | Yu, X. | 2019 | Skp2 |  | Akt | K8,K14 | Skp2-mediated ubiquitination and mitochondrial localization of Akt drive tumor growth and chemoresistance to cisplatin |
|  | Zhang, J. | 2017 | SCF |  | Akt |  | The F-box protein FBXL18 promotes glioma progression by promoting K63-linked ubiquitination of Akt |
| 2.2 RNF |  |  |  |  |  |  |  |
|  | Lee, H. J. | 2016 | RNF8 |  | Twist |  | RNF8 activates Twist by K63-linked ubiquitination, which regulates cancer cell migration and invasion and cancer metastasis |
|  | Ren, L. | 2020 | RNF8 |  | β-catenin |  | RNF8 induces β-catenin-mediated c-Myc expression and promotes colon cancer proliferation |
|  | Ren, Y. | 2020 | RNF6 |  | GR |  | RNF6 promotes myeloma cell proliferation and survival by inducing glucocorticoid receptor polyubiquitination |
|  | Zhu, J. | 2020 | RNF181 |  | ERα |  | The ubiquitin ligase RNF181 stabilizes ERα and modulates breast cancer progression |
| 2.3 TRAF |  |  |  |  |  |  |  |
|  | Choi, Y. B. | 2014 | TRAF6 |  | MCL-1 |  | HTLV-1 tax stabilizes MCL-1 via TRAF6-dependent K63-linked polyubiquitination to promote cell survival and transformation |
|  | Linares, J. F. | 2013 | TRAF6 |  | mTOR |  | K63 polyubiquitination and activation of mTOR by the p62-TRAF6 complex mediate cancer cell proliferation |
|  | Lu, W. | 2017 | TRAF6 |  | EZH2 |  | SKP2 loss destabilizes EZH2 by promoting TRAF6-mediated ubiquitination to suppress prostate cancer |
|  | Tang, Y. | 2021 | TRAF6 |  | ULK1 |  | NPM1-mA promoted TRAF6-dependent K63 ubiquitination and further maintained ULK1 stability and kinase activity to facilitate leukemic cell proliferation |
|  | Zhan, Z. | 2014 |  |  | TRAF6 |  | Autophagy facilitates TLR4- and TLR3-triggered migration and invasion of lung cancer cells through the promotion of TRAF6 ubiquitination |
|  | Zhang, J. | 2016 | TRAF6 |  |  |  | EGCG suppresses melanoma cell growth and metastasis by inhibitingTRAF6 activity |
|  | Zhang, P. | 2021 | TRAF2 |  | DYRK1A |  | K63-linked ubiquitination of DYRK1A by TRAF2 alleviates Sprouty 2-mediated degradation of EGFR to promote the growth of glioma cells |
| 2.4 CYLD |  |  |  |  |  |  |  |
|  | Johari, T. | 2018 |  | CYLD |  |  | Loss of CYLD catalytic activity potentiates its oncogenic gain of function through increased cell survival and migration. |
|  | Tauriello, D. V. | 2010 |  | CYLD | Dvl |  | Loss of the tumor suppressor CYLD enhances Wnt/beta-catenin signaling through K63-linked ubiquitination of Dvl |
|  | Xu, X. | 2020 |  | CYLD | RIPK1 |  | CYLD phosphorylation increased RIPK1 ubiquitination and provides a prosurvival signal to ATLL cells |
| 2.5 P53 |  |  |  |  |  |  |  |
|  | Guo, Y. | 2021 | TRIM31 |  | p53 |  | TRIM31 directly interacts with p53 and induces the K63-linked ubiquitination of p53 to suppress breast cancer development |
|  | Zhang, J. | 2017 | TRIM45 |  | p53 |  | TRIM45 functions as a tumor suppressor in the brain by stabilizing p53 through K63-linked ubiquitination |
|  | Zhang, X. | 2016 | TRAF6 |  | p53 |  | TRAF6 Restricts p53 Tumor Suppression function via K63-linked ubiquitination |
| 2.6 cell cycle |  |  |  |  |  |  |  |
|  | Kolapalli, S. P. | 2021 | DZIP3 |  | Cyclin D1 |  | RNA-binding RING E3-Ligase DZIP3/hRUL138 increases K63-linked ubiquitination of Cyclin D1 and stabilizes it to drive cell-cycle and cancer progression |
|  | Li, Y. | 2018 | FBW7 |  | γ-catenin |  | FBW7 suppresses cell proliferation and G2/M cell cycle transition via promoting γ-catenin K63-linked ubiquitylation |
|  | Zhang, X. | 2015 |  |  | Cyclin B1 |  | ERLIN2 facilitates K63-linked ubiquitination and stabilization of Cyclin B1 to regulate cell cycle progression associated with human breast cancer malignancy |
| 2.7 TRIM |  |  |  |  |  |  |  |
|  | Liu, K. | 2018 | TRIM9s |  | MKK6 |  | TRIM9s promotes the K63-linked ubiquitination and stabilize MKK6, which mutually stabilize TRIM9s, thus potentiating p38 signaling to synergistically suppress glioblastoma progression. |
|  | Xue, M. | 2019 | TRIM56 |  | ERα |  | TRIM56 prolongs ER alpha protein stability, through targeting ER alpha K63-linked ubiquitination to promote estrogen signaling and breast cancer proliferation |
| 2.8 metabolism |  |  |  |  |  |  |  |
|  | Lee, H. J. | 2019 | HectH9 |  | Hexokinase 2 |  | HectH9 drives glycolysis and tumor development by K63-linked ubiquitination of Hexokinase 2 (HK2) |
|  | Sun, T. | 2021 |  | PSMD14 | PKM2 |  | Deubiquitinase PSMD14 promotes ovarian cancer progression by decreasing K63-linked ubiquitination and enzymatic activity of PKM2 |
| 2.9 Others |  |  |  |  |  |  |  |
|  | Dikshit, A. | 2018 |  |  |  |  | As a K63-Ub–specific E2 enzyme, UBE2N promotes melanoma growth via MEK/FRA1/SOX10 signaling |
|  | He, Y. | 2021 |  | USP10 | PTEN | K266 | USP10 activates PTEN by preventing its K63-linked polyubiquitination mediated by TRIM25, thereby inhibiting NSCLC proliferation |
|  | Kim, D. | 2019 | Pellino-1 |  | IRAK1 |  | Pellino-1 inhibits IL-10-induced M2c macrophage polarization via K63-linked ubiquitination of IRAK1 and activation of STAT1, thereby inhibiting tumor growth |
|  | Lee, S. W. | 2015 | Skp2-SCF |  | LKB1 |  | K63-linked LKB1 polyubiquitination by Skp2-SCF ubiquitin ligase is critical for LKB1 activation and hepatocellular carcinoma growth |
|  | Meijer, I. M. | 2012 |  | AMSH | Hrs |  | EGF stimulation resulted in Hrs deubiquitination by the K63-polyubiquitin chain-specific deubiquitinating enzyme AMSH, regulating normal cellular growth |
|  | Raimondi, M. | 2019 |  | USP1 | ULK1 |  | USP1 deubiquinates K63-linked ubiquitination of ULK1 and regulates its cellular compartmentalization and autophagy to regulate tumor growth |
|  | Shao, N. | 2021 | FBXO42 |  | ROCK2 |  | GASC1 promotes hepatocellular carcinoma progression by inhibiting the FBXO42-mediated K63-linked poly-ubiquitination degradation of ROCK2 |
|  | Sun, L. | 2021 | NEDD4 |  | IGPR-1 |  | NEDD4 mediates K63-linked polyubiquitination of IGPR-1 leading to its lysosomal-dependent degradation and inhibition of tumor growth |
|  | Wang, J. Y. | 2017 | Skp2 |  | MTH1 |  | Skp2-mediated K63-linked polyubiquitination stabilizes MTH1 and promotes survival of melanoma cells upon oxidative stress |
|  | Wu, H. T. | 2016 | HectH9 |  | HAUSP | K443 | HectH9-mediated K63-polyubiquitination of HAUSP deubiquitinates HIF-1α and dictates H3K56 acetylation promoting hypoxia-induced tumour progression |
| 3.1 breast cancer |  |  |  |  |  |  |  |
|  | Niu, T. | 2021 | TRAF6 |  | AKT |  | Uev1A/Ubc13-AKT-CT45A axis promotes breast cancer cell migration and EMT signaling |
|  | Ray, D. M. | 2010 |  |  |  |  | Lysine 63-linked ubiquitination is important for arachidonic acid-induced cellular adhesion and migration |
|  | Tan, P. | 2018 | RNFT1 |  | PDCD10 | K179 | TRIM59 blocks RNFT1-mediated K63 ubiquitination of PDCD10 to stabilize it and promote breast cancer motility |
| 3.2 lung cancer |  |  |  |  |  |  |  |
|  | He, Z. | 2016 | TRAF6 |  |  |  | TRAF6 knockdown promotes the apoptosis and inhibits the invasion of human lung cancer cells |
|  | Kim, E. | 2017 |  |  | TRAF4 |  | The K63-linkage poly-ubiquitinated TRAF4 promotes lung cancer aggressiveness by modulating tumor microenvironment in normal fibroblasts |
|  | Li, Y. | 2018 | TRIM37 |  | TRAF2 |  | TRIM37 mediates K63-linked ubiquitination of TRAF2 and activates the NF-κB pathway, which promotes the aggressiveness of non-small-cell lung cancer cells |
| 3.3 nervous system cancer |  |  |  |  |  |  |  |
|  | Li, X. | 2017 | TRAF6 |  | Beclin1 |  | CaMKII phosphorylates Beclin 1 to promote its K63-linked ubiquitination and to activate autophagy and differentiation of neuroblastoma cells |
|  | Wald, J. H. | 2017 | Nrdp1 |  | Dvl |  | Nrdp1-mediated K63-linked polyubiquitination of Dvl suppress the migration in glioblastoma |
|  | Wang, L. | 2017 | Nedd4-1 |  | Rap2a |  | Nedd4-1 regulaties the migration and invasion of glioma cells via mediating Rap2a K63-linked ubiquitination |
| 3.4 gastric cancer |  |  |  |  |  |  |  |
|  | Li, X. | 2020 |  |  | LDHA |  | LDHA succinylation inhibits the degradation of K63-ubiquitinated LDHA by lysosomal pathway, promoting GC invasion and proliferation |
| 4.1 breast cancer |  |  |  |  |  |  |  |
|  | Lin, Y. | 2014 | TRAF6 | USP17 | AEP |  | TRAF6 and USP17 regulate AEP K63-linked polyubiquitin, affecting tumor invasion and metastasis |
|  | Gong, Z. | 2021 |  | OTUD7B | LSD1 | K226, K277 | OTUD7B deubiquitinates LSD1 to govern breast cancer metastasis |
|  | Tan, P. | 2019 |  | RNFT1 | PDCD10 |  | TRIM59 deficiency curtails breast cancer metastasis through regulating RNFT1-induced K63 polyubiquitination of PDCD10 |
|  | Wu, X. | 2014 |  |  |  |  | Ubiquitin-conjugating enzyme Ubc13 controls breast cancer metastasis through a TAK1-p38 MAP kinase cascade |
| 4.2 digestive cancer |  |  |  |  |  |  |  |
|  | Wu, H. | 2019 | TRAF6 |  | LC3B |  | TRAF6 inhibits colorectal cancer metastasis through regulating K63-linked polyubiquitination of LC3B |
|  | Wu, Z. | 2018 |  |  |  |  | Uev1A-Ubc13 promotes colorectal cancer metastasis through regulating CXCL1 expression via NF-кB activation |
|  | Zhu, Y. | 2019 | RNF8 | Trabid | Twist1 |  | Trabid inhibits hepatocellular carcinoma growth and metastasis by cleaving RNF8-induced K63 ubiquitination of Twist1 |
| 4.3 Others |  |  |  |  |  |  |  |
|  | Kuang, J. | 2020 | RNF8 |  | Slug |  | RNF8 promotes Epithelial-Mesenchymal Transition in lung cancer cells via K63-linked polyubiquitin and stabilization of Slug |
|  | Luo, Z. | 2016 | TRAF6 |  | Basigin |  | TRAF6 regulates melanoma invasion and metastasis through ubiquitination of Basigin |
| 5.1 TNF/TNFR |  |  |  |  |  |  |  |
|  | Fukuyo, Y. | 2009 |  |  | Daxx | K122 | K63-linked polyubiquitination of Daxx functions as a molecular switch to initiate and amplify the stress kinase response in the TNF-alpha induced apoptotic response |
|  | Hou, X. | 2013 |  | USP4 | RIP1 |  | USP4 promotes TNF-α-induced apoptosis by K63-linked deubiquitination of RIP1 in head and neck squamous cell carcinoma |
|  | Law, J. | 2018 | TRAF2 |  | MOAP-1 |  | RACK1 brings the E3 ligase TRAF2 to MOAP-1 to undergo a K63-dependent ubiquitination and drive Bax-driven apoptosis |
|  | Wo, L. | 2016 |  | CYLD | RIP1 |  | Knockdown of miR-182 promotes apoptosis via regulating CYLD mediated RIP1 K63 deubiquitination in breast cancer |
| 5.2 EBV |  |  |  |  |  |  |  |
|  | Gain, C. | 2020 |  |  | EBNA3C |  | Susceptibility of proteasomal inhibitors mediated cell death in EBNA3C expressing B-cells was found via apoptosis. |
|  | Li, L. | 2012 | TRAF2 |  | p53 |  | LMP1 disrupts p53-induced apoptosis through modulating TRAF2-mediated K63-linked ubiquitination of p53 |
| 5.3 mitochondrial |  |  |  |  |  |  |  |
|  | Jing, B. | 2018 |  |  |  |  | Vorinostat and quinacrine have synergistic effects in T-cell acute lymphoblastic leukemia through regulating K63-linked ubiquitination of the mitochondria. |
|  | Lee, H. J. | 2019 | HectH9 |  | HK2 | K104 | K63-linked ubiquitination of HK2 by HectH9 controls tumor metabolism and apoptosis |
| 5.4 Others |  |  |  |  |  |  |  |
|  | He, Y. M. | 2021 | TRIM25 |  | PTEN | K266 | TRIM25 mediates K63-linked ubiquitination of PTEN to activate the AKT/mTOR signaling and promote NSCLC tumor growth and inhibit apoptosis |
|  | Akiyama, H. | 2019 |  | USP9X | FLT3-ITD |  | Inhibition of USP9X promotes FLT3-ITD K63-linked polyubiquitination to induce apoptosis in FLT3-ITD-positive AML cells |
|  | Kapuria, V. | 2010 |  | USP9x, USP5, USP14, UCH37 |  |  | WP1130, a small-molecule DUB inhibitor of USP9x, USP5, USP14, and UCH37, triggers aggresome formation and tumor cell apoptosis |
|  | Li, Y. | 2013 | HECTD3 |  | caspase-8 | K215 | HECTD3 facilitates cancer cell survival and inhibits apoptosis by promoting K63-linked polyubiquitination of caspase-8 |
|  | Sun, H. | 2011 |  |  | Bcr-Abl |  | Bcr-Abl ubiquitination and Usp9x inhibition by WP1130 block kinase signaling and promote CML cell apoptosis. |
|  | Wu, P. | 2014 | cIAP | CYLD | RIP1 |  | Selenite caused CYLD upregulation via LEF1 and cIAP downregulation, contributed to the degradation of ubiquitin chains on RIP1 and subsequent caspase-8 activation and apoptosis |
| 6.1 T cell |  |  |  |  |  |  |  |
|  | Martinez-Forero, I. | 2013 |  |  | TRAF2 |  | T Cell Costimulation with Anti-CD137 Monoclonal Antibodies Is Mediated by K63–Polyubiquitin-Dependent Signals from Endosomes |
|  | Ni, X. | 2019 | TRAF6 |  | FOXP3 | K262 | TRAF6 directs FOXP3 localization and facilitates regulatory T-cell function through K63-linked ubiquitination |
| 6.2 Th9 |  |  |  |  |  |  |  |
|  | Pei, S. | 2021 | BFAR |  | TGFβR1 | K268 | BFAR mediates K63-linked ubiquitination of TGFβR1 and Th9 differentiation, thereby promoting Th9-mediated cancer immunotherapy |
|  | Rivera Vargas, T. | 2017 |  |  | PU.1 |  | Selective degradation of PU.1 via K63 ubiquitination during autophagy represses the differentiation and antitumour activity of T(H)9 cells |
| 6.3 MDSC |  |  |  |  |  |  |  |
|  | Song, G. | 2021 | TRAF6 |  | STAT3 |  | TRAF6 participates in promoting the immunosuppressive function of MDSCs by mediating K63-linked polyubiquitination of STAT3 |
|  | Xin, J. | 2017 |  |  | STAT3 |  | Silencing p66a could promote MDSC accumulation, differentiation, and activation through K63 ubiquitination of STAT3 |
| 6.4 Others |  |  |  |  |  |  |  |
|  | He, H. | 2021 |  |  | TRAF6 |  | CagA interacts with SHP-1, which promoted the recruitment of SHP-1 to TRAF6 and inhibited the K63-linked ubiquitination of TRAF6, to inhibit the expression of proinflammatory cytokines during H. pylori infection. |
|  | Takahashi, M. | 2021 | LMO7 |  | STING |  | DAPK3 promotes STING activation by phosphorylating the E3 ligase LMO7, STING K63-linked polyubiquitination, recruitment of TBK1 and driving tumor-intrinsic immunity |
|  | Wang, Y. | 2020 |  | USP22 | CSN5, PD-L1 |  | USP22 regulates PD-L1 degradation by removing K63-linked ubiquitin of PD-L1 or CSN5, which plays an important role in PD-L1 mediated immune evasion |
|  | Wu, S. | 2019 |  |  | TBK1 |  | HER2 recruits AKT1 to prevent TBK1 K63-linked ubiquitination, which disrupts STING signalling and suppresses antiviral defence and antitumour immunity |
| 7.1 TRAF2/5 |  |  |  |  |  |  |  |
|  | Dai, T. | 2015 |  |  | TRAF2, RIP, NEMO |  | GOLPH3 promoted K63-linked polyubiquitination of TRAF2, RIP, NEMO and sustained the activation of NF-κB in HCC cells |
|  | Gudi, R. | 2009 |  |  | TRAF2 |  | Siva-1 Inhibits K63-Polyubiquitination-Mediated Activation of NF-κB by TRAF2 |
|  | Yu, C. | 2018 | TRIM31 |  | TRAF2 |  | TRIM31 confers gemcitabine resistance in pancreatic cancer by promoting K63-linked polyubiquitination of TRAF2 and sustains the activation of NF-κB |
|  | Zhou, A. Y. | 2013 | cIAP1/cIAP2/TRAF2 |  | IKKε | K30, K401 | cIAP1/cIAP2/TRAF2 E3 ubiquitin ligase complex mediates K63-linked polyubiquitination of IKKε, which promotes malignant transformation through NF-Κb activation |
|  | Boulabiar, M. | 2011 |  |  | TRAF5 |  | E2 potentiates TNF-induced NFκB signaling mediated by K63-linked ubiquitination mediated TRAF5 activation |
| 7.1 IKKβ/IKK |  |  |  |  |  |  |  |
|  | Yang, Y. | 2016 | cIAP1/2 |  | BCL10 | K31, K63 | cIAP1/2 attach K63-linked polyubiquitin chains on themselves and on BCL10, resulting in the recruitment and activation of IKK |
|  | Gallo, L. H. | 2014 |  |  | IKKβ | K147 | Inhibition of the UBC13-UEV1A complex responsible for K63-linked ubiquitination of IKKβ and responsible for STAT3 activation |
|  | Meyer, A. N. | 2018 |  |  | IKKβ | K147 | K63-linked ubiquitination occurs in other kinases at sites homologous to K147 in IKKβ, which serves as an oncogenic driver in melanoma |
|  | Wang, Y. | 2016 | pVHL |  | IKKβ |  | pVHL mediates K63-linked ubiquitination of IKKβ, leading to IKKβ inactivation |
| 7.1 RIP1 |  |  |  |  |  |  |  |
|  | Gong, H. | 2013 |  |  | TRAF2, RIP1 |  | Silencing miR-138 promoted K63-linked polyubiquitination of TRAF2 and RIP1 and sustained NF-κB activation in esophageal squamous cell carcinoma |
|  | Kim, S. W. | 2012 |  |  | TRAF2, RIP1 |  | MicroRNAs miR-125a and miR-125b activate the NF-κB pathway by targeting TNFAIP3 and regulating K-63 ubiquitination of TRAF2 and RIP1 |
|  | Puliyappadamba, V. T. | 2013 |  |  | RIP1 |  | The EGFRWT-EGFRvIII-RIP1 interplay via K63-linked ubiquitination of RIP1 may regulate oncogenicity of GBM |
|  | Song, L. | 2012 |  |  | TRAF2, RIP, NEMO |  | FLOT1 promotes K63-linked polyubiquitination of the signaling intermediaries TRAF2, RIP, NEMO and sustained the activation of NF-κB in esophageal squamous cell carcinoma cells |
|  | Yan, X. Y. | 2017 |  |  | RIP1 |  | p62 mediates cisplatin resistance through promoting K63-linked ubiquitination of RIP1 and activating RIP1-NF-κB pathway in human ovarian cancer cells |
| 7.4 CYLD |  |  |  |  |  |  |  |
|  | Almeida, S. | 2008 |  | CYLD | TRAF2, TRAF6 |  | CYLD D681G mutation is unable to cleave K63-linked polyubiquitin chains, having a significant reduced ability to inhibit TRAF2- and TRAF6-mediated NF-kappaB activation |
|  | An, J. | 2008 |  | CYLD |  |  | The HPV-encoded E6 protein prolong hypoxia-induced NF-κB activation targeting CYLD K63 deubiquitinase |
| 7.5 Others |  |  |  |  |  |  |  |
|  | Chen, M. | 2020 |  | USP14 | p100/p52 | K332, K338, K341 | TRIM14 Promotes Noncanonical NF-κB Activation by Modulating p100/p52 Stability via Selective Autophagy |
|  | Guo, M. | 2019 |  |  | MSR1 | K27 | Triggering of MSR1 mediated through K63 polyubiquitylation in IL-4-activated macrophages leads to enhanced JNK activation |
|  | Ji, J. | 2021 | TRIM22 |  | IKKγ |  | TRIM22 activates NF-κB signaling in glioblastoma by K63-linked ubiquitination of IKKγ |
|  | Yu, X. | 2021 | RNF138 |  | MYD88L265P |  | RNF138 catalyzed K63-linked nonproteolytic polyubiquitination of MYD88L265P, resulting in elevated NF-κB activation |
| 8.1.1 NHEJ |  |  |  |  |  |  |  |
|  | Yang, Y. | 2020 |  | USP38 | HDAC1 |  | The Deubiquitinase USP38 Promotes NHEJ Repair through Regulation of HDAC1 Activity and Regulates Cancer Cell Response to Genotoxic Insults |
|  | Zhang, Q. | 2016 | FBXW7 |  | XRCC4 | K296 | FBXW7 Facilitates Nonhomologous End-Joining via K63-Linked Polyubiquitylation of XRCC4 |
| 8.1.2 HR |  |  |  |  |  |  |  |
|  | Ali, M. A. M. | 2018 |  |  |  |  | RYBP negatively regulates HR repair by competing for K63-ubiquitin chain binding |
|  | Wu, J. | 2012 | Skp2 |  | NBS1 | K735 | Skp2 E3 ligase integrates ATM activation and homologous recombination repair by ubiquitinating NBS1 |
|  | Zhu, B. | 2015 |  |  | FANCG | K182, K258, K347 | K63-linked ubiquitination of FANCG is required for its association with the Rap80-BRCA1 complex to modulate homologous recombination repair |
| 8.1.3 Other DSBR |  |  |  |  |  |  |  |
|  | Abu-Odeh, M. | 2014 | ITCH |  | WWOX | K274 | WWOX expression regulated by ITCH-mediated K63-linked ubiquitination affect ATM activation and DNA repair. |
|  | Liu, P. | 2018 |  |  |  |  | K63-linked polyubiquitin chains bind to DNA to facilitate DNA damage repair |
|  | Metcalf, J. L. | 2014 | SOCS1 |  | VHL | K196 | K63-ubiquitylation of VHL by SOCS1 mediates DNA double-strand break repair |
|  | Wu, M. | 2017 |  | USP19 | HDAC1/2 |  | USP19 deubiquitinates HDAC1/2 to regulate DNA damage repair and control chromosomal stability |
|  | Zhi, H. | 2020 | RNF8 |  |  |  | Tax activates RNF8 to assemble nuclear K63-pUbs disrupting DDR signaling and DSB repair in ATL cells |
| 8.2 Others |  |  |  |  |  |  |  |
|  | Fernández-Majada, V. | 2016 |  | CYLD | p53 |  | CYLD is a deubiquitinase facilitating DNA damage-induced p53 activation and suggest that regulation of p53 responses to genotoxic stress |
|  | Vujanovic, M. | 2017 |  |  | PCNA |  | UBC13 mediates K63-linked PCNA ubiquitination and regulates DNA damage-induced replication fork slowing |
| 9.1 chemotherapeutic drug |  |  |  |  |  |  |  |
|  | Delbue, D. | 2020 | XIAP |  |  |  | Expression of nuclear XIAP associates with cell growth and drug resistance via k63-linked ubiquitination |
|  | Li, Z. | 2015 | TRAF6 |  | MCL1 |  | IRAK1/4 signaling activated the E3 ubiquitin ligase TRAF6, increasing K63-linked ubiquitination of MCL1 to regulate chemotherapy sensitivity |
|  | Qu, C. | 2018 |  | USP8 | TRAF6 |  | SMO/TRAF6/AKT is highly relevant in the biology of DLBCL and is involved in doxorubicin resistance. |
|  | Shi, W. N. | 2017 |  |  | RXRα |  | SphK2 contributes to ATRA resistance in colon cancer through rapid degradation of cytoplasmic RXRα by K48/K63-linked polyubiquitination |
| 9.2 targeted drug |  |  |  |  |  |  |  |
|  | Gong, K. | 2020 | TRIM32 |  | TBK1 |  | EGFR inhibition triggers an adaptive response by co-opting antiviral signaling pathways in lung cancer |
|  | Li, Y. | 2018 | RBX1 |  | POLR2A |  | RBX1 activates POLR2A by the K63-linked ubiquitination, affecting prostate cancer sensitivity to inhibition of RNA polymerase II |
|  | Marx, C. | 2010 | c-Cbl | USP9x | ErbB2 |  | ErbB2 Trafficking and Degradation Associated with K48 and K63 Polyubiquitination |
|  | Nunes, J. | 2016 | c-Cbl |  | Her2 |  | ATG9A loss in trastuzumab resistant cells allowed Her2 to escape from lysosomal targeted degradation through K63 poly-ubiquitination via c-Cbl |
|  | Zhu, G. | 2021 | TRIM15 | CYLD | ERK | K168, K302 | TRIM15 and CYLD regulate ERK activation via lysine-63-linked polyubiquitination |

**Supplementary Table 2. Characteristics of the drugs used in cancer treatment with the regulation of K63-linked ubiquitination.**

|  | **Drug display** | **Mechanism of action** | **Stage of drug research** |
| --- | --- | --- | --- |
| **Chemotherapeutic drug** | doxorubicin and paclitaxel | Doxorubicin acts by binding to DNA-associated enzymes, it can intercalate the base pairs of the DNA's double helix. Paclitaxel stabilizes microtubules and arrests somatic cell mitosis at the G2/M stage of the replication | clinical |
|  | ABT-737 and vincristine with IRAK1/4 inhibitor | ABT-737 is a BH3 inhibitor that mimics the BH3 domain of preapoptotic proteins, increasing mitochondrial outer membrane permeability and releasing Cyt-C from the mitochondrial membrane space into the cytoplasm. ABT-737 then binds to the cytoplasmic factor APAF1, promotes its oligomerization to form apoptotic bodies and activates caspase 9, which further activates other downstream caspase family proteins and ultimately promotes tumor cell apoptosis. By binding to tubulin, Vincristine prevents the assembly of microtubules and prevents the formation of spindle cells, thus stopping cell division and inducing apoptosis of tumor cells. | preclinical (ABT-737), clinical (vincristine) |
|  | doxorubicin | Doxorubicin acts by binding to DNA-associated enzymes, it can intercalate the base pairs of the DNA's double helix. | clinical |
|  | ATRA | All-trans retinoic acid (ATRA), one of the metabolites of vitamin A, terminally differentiates immature myelocytic tumor cells, resulting in death of the tumors cells. | clinical |
| **Targeted drug** | erlotinib (EGFR-TKI) and anifrolumab (IFN inhibitor) | EGFR-TKIs inhibit the EGFR tyrosine kinase domain in an ATP-competitive and reversible manner, which inhibited the growth and proliferation of tumor cells and promoted cell apoptosis. Anifrolumab is a monoclonal antibody directed against IFNAR that inhibits the binding of Type I IFNs to its receptor. | clinical |
|  | α-amanitin-based ADC (RNAP2 inhibitor) | Inhibition of POLR2A with α-amanitin-based ADC selectively suppresses the proliferation, survival and tumor growth of CRPC cells harboring deletion of 17p. | preclinical |
|  | bortezomib/PS341 and geldanamycin (GA) | Bortezomib (PS-341), a reversible and selective proteasome inhibitor, has anticancer activity by targeting threonine residues to effectively inhibit the 20S proteasome, thereby disrupting the cell cycle, inducing apoptosis, and inhibiting the nuclear factor NF-κB. Geldanamycin, a benzoquinone ansamycin antibiotic, exhibits anticancer activity by inhibiting hsp90-chaperone function and inducing G1 phase arrest of the cell cycle. | clinical (bortezomib/PS341), preclinical (geldanamycin) |
|  | trastuzumab | Trastuzumab is a recombinant DNA-derived humanized monoclonal antibody that specifically acts on the extracellular site of human epidermal growth factor receptor-2 (HER2) and inhibits the proliferation of HER2-overexpressing tumor cells | clinical |
|  | PLX4032 (Vemurafenib) | Vemurafenib (PLX4032) was developed as a low-molecular-weight molecule for the inhibition of the mutated serine-threonine kinase BRAF, and it selectively binds to the ATP-binding site of BRAF V600E kinase and inhibits its activity. | clinical |
